# Supplementary material for: Women’s extreme seclusion during menstruation and children’s health in Nepal
Source: PLOS Glob Public Health. 2022 Jul 20;2(7):e0000355. doi: 10.1371/journal.pgph.0000355 (PMC10021664; doi:10.1371/journal.pgph.0000355)
Supplement: S1 File — (DOCX) [file pgph.0000355.s002.docx]

**S2 Stata Codes**. Replication Codes for Joshi S & Acharya Y. Women's Extreme Seclusion during Menstruation and Children's Health in Nepal (PLOS Global Public Health, 2022).

global root "C:\Users\yua36\Desktop\Chhaupadi" //This is where the analytic database sits and where the results are saved

ssc install table1_mc

use "$root\chwmhh.dta", clear

count //no of observations is 6641, which matches the CH file

***define globals**

global outcome1 waz haz //anthropometric outcomes

global outcome2 dirr difbreath //health outcomes

global childvar1 i.girl cage dd misdd //child-level covariates for anthropometric outcomes

global childvar2 i.girl cage //child-level covariates for health outcomes

global momvar mage agemar i.edu //mother-level covariates

global hhvar i.sanitation i.water i.wealth i.twater i.urban i.eth4 //household-level covariates

***create necessary variables**

*outcomes

clonevar haz=HAZ2 //who z-score

replace haz=. if HAZFLAG==1

clonevar waz=WAZ2 //who z-score for weight

replace waz=. if WAZFLAG==1

gen dirr=(CA1==1) //diarrhea in last 2 wks

replace dirr=. if CA1>2

gen difbreath = 0 //difficulty breathing in 2 wks

replace difbreath = 1 if CA17 == 1

replace difbreath = . if CA17 >2

*main independent variables

clonevar chhau = UN16AA // chhauhut

replace chhau = 1 if UN16AA == 1

replace chhau = 0 if UN16AA == 2

replace chhau = . if UN16AA == 9

clonevar shed = UN16AC // cowshed

replace shed = 1 if UN16AC == 1

replace shed = 0 if UN16AC == 2

replace shed = . if UN16AC == 9

label variable chhau "whether stayed in Chhau-Shed"

label define yesno 0 "No" 1 "Yes"

label values chhau yesno

label variable shed "whether stayed in Cow-Shed"

label values shed yesno

gen combine=0 // overall exposure

replace combine=1 if chhau==1 | shed==1

label variable combine "whether stayed in shed or hut"

label values combine yesno

*covariates

gen girl=(HL4==2) //gender (1= girl, 0 = boy)

label variable girl "sex"

label define malefemale 0 "boys" 1 "girls"

label values girl malefemale

gen cage = CAGE // children's age in month

clonevar mage=WB4 //mother's age

clonevar edu=welevel1 //education

recode edu (0=1) (2=2) (5=3) (8=4)

clonevar agemar=WAGEM //age at first marriage

replace agemar=. if agemar==97

clonevar province=HH7c //province

gen urban=(HH6==1) // Urban/rural (urban = 1, rural = 0)

gen sanitation = . //sanitation 1 = access to flush toilet; 0 = no)

replace sanitation = 1 if (WS11==11 | WS11==12)

replace sanitation = 0 if WS11!=11 & WS11!=12 & WS11!=.

gen water = . // driking water access (1= pipied into dwelling and property; not piped into dwellings)

replace water = 1 if (WS1==11 | WS1==12)

replace water = 0 if WS1!=11 & WS1!=12 & WS1!=.

gen twater = 0 // Treat water to make safer for drinking (yes =1, NO=0)

replace twater = 1 if WS9 == 2

replace twater = . if WS9 >=8

fre windex5 // wealth index

clonevar wealth = windex5

replace wealth = . if wealth == 0

fre wealth

*diet diversity

gen staples = .

replace staples = 1 if BD8C == 1 | BD8B == 1

replace staples = 0 if BD8C == 2 & BD8B == 2

gen legumes = .

replace legumes = 1 if BD8M== 1

replace legumes = 0 if BD8M == 2

gen dairy = .

replace dairy = 1 if BD8A == 1 | BD7E == 1 | BD8N== 1

replace dairy = 0 if BD8A == 2 & BD7E == 2 & BD8N== 2

gen flesh = .

replace flesh = 1 if BD8J == 1 | BD8I == 1

replace flesh = 0 if BD8J == 2 & BD8I == 2

gen egg = .

replace egg = 1 if BD8K == 1

replace egg = 0 if BD8K == 2

gen vitaveg = .

replace vitaveg = 1 if BD8D == 1 | BD8E == 1 | BD8F == 1 | BD8G == 1

replace vitaveg = 0 if BD8D == 2 & BD8E == 2 & BD8F == 2 & BD8G == 2

gen otherveg = .

replace otherveg = 1 if BD8H == 1

replace otherveg = 0 if BD8H == 2

egen dd=rowtotal( staples- otherveg)

count if staples==. & legumes==. & dairy ==. & flesh ==. & egg==. & vitaveg==. & otherveg==.

gen misdd=0

replace misdd=1 if staples==. & legumes==. & dairy ==. & flesh ==. & egg==. & vitaveg==. & otherveg==.

// ethnicity

recode HC2 (2=201 "Hill Brahmin")(1 14 20 = 202 "Chhetri Hill") (27 48 = 203 "Tarai/Madeshi Brahmin Chhetri") ///

(93 55 63 96 64 47 56 31 34 28 49 26 44 19 58 38 69 43 59 86 30 25 37 16 9 = 204 "Tarai/Madeshi Other Castes") ///

(50 82 12 73 8 15 = 205 "Hill Dalit") (17 83 41 72 23 33 40 87 22 39 = 206 "Tarai/Madesh Dalit") ///

(6 = 207 "Newar") (91 97 95 54 62 90 46 71 45 66 85 29 11 79 74 98 84 32 76 80 13 3 42 94 88 10 97 24 36 81 5 68 60 65 89 = 208 "Hill/Mountain Janajati") ///

(21 61 57 53 78 18 35 52 67 4 = 209 "Terai Janjati") ///

(75 7 = 210 "Muslim") (126 = 211 "Dalit Others") (127 = 212 "Janajati Others") (128 = 213 "Tarai Others") ///

(51 104 105 108 111 112 114 116 119 121 996 = 214 "Others"), gen(eth14)

recode eth14 (201 202 203 = 1 "Brahmin/Chhetri") (205 206 211 = 2 "Dalit") (208 212 209 = 3 "Janjati") ( 207 204 210 213 214 = 5 "Others"), gen(eth4)

*marknomiss

mark nomiss

markout nomiss $childvar1 $momvar $hhvar $outcome1 $outcome2

***Table 1**

preserve

keep if nomiss==1

table1_mc, by(combine) ///

vars( ///

girl bin %4.2f \ ///

cage contn %4.2f \ ///

mage contn %4.2f \ ///

agemar contn %4.2f \ ///

edu cat %4.2f \ ///

sanitation bin %4.2f \ ///

water bin %4.2f \ ///

twater bin %4.2f \ ///

wealth cat %4.2f \ ///

urban bin %4.2f \ ///

eth4 cat %4.2f \ ///

waz contn %4.2f \ ///

haz contn %4.2f \ ///

dirr bin %4.2f \ ///

difbreath bin %4.2f \ ///

) ///

nospace percent onecol missing total(before) ///

saving("$root\table 1.xlsx", replace)

ttest dd if misdd==0, by(combine) //need to do the t-test separately for diet diversity

restore

***set survey weights**

svyset PSU [pweight=chweight], strata(stratum)

***regressions - OLS for anthropometric outcomes**

foreach x in $outcome1 {

svy: reg `x' combine $childvar1 $momvar $hhvar i.province if nomiss

est store `x'_m1

svy: reg `x' shed $childvar1 $momvar $hhvar i.province if nomiss

est store `x'_m2

svy: reg `x' chhau $childvar1 $momvar $hhvar i.province if nomiss

est store `x'_m3

}

*regressions - run logit and report ORs for binary outcomes

foreach x in $outcome2 {

svy: logit `x' combine $childvar2 $momvar $hhvar i.province if nomiss, or

est store `x'_m4

svy: logit `x' shed $childvar2 $momvar $hhvar i.province if nomiss, or

est store `x'_m5

svy: logit `x' chhau $childvar2 $momvar $hhvar i.province if nomiss, or

est store `x'_m6

}

***Table 2**

esttab haz_m1 waz_m1 haz_m2 waz_m2 haz_m3 waz_m3, cells(b(star fmt(3)) se(par fmt(2))) star(* 0.10 ** 0.05 *** 0.01) r2

***Table 3**

esttab dirr_m4 difbreath_m4 dirr_m5 difbreath_m5 dirr_m6 difbreath_m6, keep(combine shed chhau) eform cells(b(star fmt(3)) p(par fmt(4))) star(* 0.10 ** 0.05 *** 0.01) r2

*****End of analysis*****
